# Perceived professional competence and workplace violence management ability among nursing students: a cross-sectional study

**DOI:** 10.1186/s12912-026-04324-5

**Published:** 2026-01-20

**Authors:** Ahmet Erol, Zeliha Cengiz, Uğur Öner

**Affiliations:** 1 Department of Fundamentals Nursing, Faculty of Health Sciences, Batman University, Batman, Türkiye; 2 Department of Fundamentals Nursing, Faculty of Nursing, İnönü University, Malatya, Türkiye

**Keywords:** Nursing students, Professional competence, Self-efficacy, Workplace violence

## Abstract

**Background:**

Nursing students frequently face workplace violence during clinical practice, which may affect their professional competence and ability to manage such situations. Although workplace violence in nursing education has been studied, the relationship between students’ professional competence and their violence management competence remains insufficiently explored. Therefore, this study aimed to examine this relationship in clinical practice.

**Methods:**

This descriptive-correlational study used a convenience sampling method and included 223 nursing students from a public university between January and May 2024. Data were collected using the Student Information Form, the Competency Inventory for Nursing Students, and the Management of Workplace Violence Competence Scale. Statistical analyses included descriptive statistics, Pearson correlation analysis, linear regression analysis, and group comparisons using ANOVA and independent t-tests.

**Results:**

A moderate positive correlation was found between professional competence and violence management competence. The mean age of the students was 21.16 years; most were female (65%) and in their third or fourth year of study. Professional competence and violence management scores were slightly above the theoretical midpoints (M = 231.65, SD = 37.14; M = 98.23, SD = 13.48). Nearly half of the students (45.8%) reported exposure to workplace violence, most commonly verbal and psychological. Higher competence levels were descriptively observed based on self-reported self-confidence and problem-solving ability. Professional competence significantly predicted violence management competence (β = 0.388, R² = 0.154, *p* < 0.001).

**Conclusions:**

Professional competence and violence management are interrelated. Self-confidence, problem-solving ability, motivation, and passion for nursing showed descriptive associations with both competence measures.

## Introduction

Clinical practice is an essential part of nursing education, providing students with the opportunity to enhance fundamental clinical skills by applying theoretical knowledge in practical healthcare environments. Additionally, it enables students to develop nursing skills, clinical understanding, and professionalism [1]. Effective clinical teaching is crucial for improving nursing students’ competencies in clinical practice [2]. Despite its vital importance in nursing education, students face various challenges in clinical practice. Such challenges include exposure to violence, material shortages, non-nursing responsibilities, high numbers of students in clinical practice, and difficulties in translating theoretical knowledge into practice [3–5].

Violence is a factor that is commonly encountered in the healthcare sector, causing problems across various aspects [3, 4]. Nursing students who actively participate in healthcare settings frequently face workplace violence [3, 6–8]. Nursing students are more vulnerable to violence in clinical settings. This is generally associated with their younger age and limited clinical experience compared to other staff members [6, 9]. One study reported that nursing students are often exposed to violence in clinical practice, with verbal and psychological violence being the most common forms [7]. Another study indicates that nursing students encounter peer violence, and their stress levels increase due to the verbal and psychological violence they are exposed to [10]. A qualitative study reported that violence experienced by nursing students during clinical practice included personal humiliation, disrespect, and hostility from colleagues. Students also reported experiencing violence from nurses and their instructors for the tasks they are unable to perform. In the same study, students expressed feelings of confusion, shame, fear, anger, sadness, humiliation and disappointment after being exposed to violence [9].

The cultural and healthcare context in Türkiye may influence nursing students’ experiences of workplace violence. High patient density, heavy workloads, overcrowded facilities, and hierarchical clinical structures may increase the risk of aggressive interactions [6, 7, 10]. In addition, cultural norms related to authority, communication styles, and expectations from healthcare workers may affect both the occurrence of violence and students’ willingness to report such incidents [3, 4].

Violence against nursing students is a critical issue with substantial negative consequences for students’ wellbeing, learning processes, and future professional practice [6, 9, 10]. Professional competence, which encompasses the knowledge, skills, and attitudes required for effective nursing care, plays a central role in helping students manage both clinical responsibilities and challenging situations such as workplace violence [11, 12]. Clinical practice provides a vital opportunity for students to translate theoretical knowledge into hands-on experience and to develop this competence through real patient care [13]. However, when professional competence is insufficiently developed, students may experience greater difficulty in managing interpersonal conflicts and may become more vulnerable to violence from peers, instructors, patients, and their relatives [6, 7]. Therefore, examining nursing students’ levels of professional competence alongside their experiences of workplace violence and their ability to manage such incidents is essential for understanding how clinical safety and professional development intersect in nursing education. Recent qualitative evidence further supports this association by showing that exposure to workplace violence negatively affects nursing students’ emotional wellbeing, academic engagement, and professional commitment [14].

Previous studies have widely examined nursing students’ exposure to workplace violence and its psychological consequences such as stress, fear and learning difficulties [3, 4, 6, 7, 15]. Other studies have focused on nursing students’ professional competence and its association with self-efficacy, clinical learning environments and academic performance [16–18]. However, these two areas have mostly been investigated separately. There is still limited evidence on the direct relationship between nursing students’ professional competence and their violence management competence in clinical practice. Therefore, this study aims to contribute to the existing literature by exploring this relationship. In addition to the academic gap, recurring challenges observed during clinical education regarding nursing students’ exposure to workplace violence also motivated this study. Although workplace violence and professional competence have been widely examined in nursing education, these constructs have largely been studied in isolation across different contexts. By integrating professional competence with workplace violence management competence within a self-efficacy framework, the present study addresses an underexplored intersection with direct relevance for clinical education and student preparedness across diverse healthcare systems.

From a theoretical perspective, the relationship between professional competence and violence management competence can be explained through Bandura’s Self-Efficacy Theory [19]. According to this theory, individuals with higher self-efficacy feel more capable of managing difficult situations. In nursing education, professional competence enhances self-efficacy by strengthening students’ clinical skills and confidence. Therefore, students with higher professional competence may feel better equipped to manage violent situations encountered during clinical practice.

### Aim of the study

This study aimed to determine nursing students’ exposure to violence during clinical practice, their levels of professional competence, their violence management competence, and the relationships among these variables.

**Research Questions**

1. What are nursing students’ levels of professional competence and violence management competence?
2. Is there a relationship between professional competence and violence management competence?
3. Which factors are associated with these competence levels among nursing students?

## Methods

### Design

The study was conducted using a descriptive-correlational design.

### Population and sample

The study population consisted of 400 nursing students enrolled in a public university between January and May 2024. The sample size was calculated as a minimum of 197 students, with a 95% confidence level, 5% margin of error, and statistical power of 95%. The study was completed with 223 students. A convenience sampling method was used to recruit students who met the inclusion criteria and agreed to participate.

During data collection, 265 students were approached in classroom settings; students who declined participation or returned incomplete questionnaires were excluded, and data from 223 fully completed questionnaires were included in the analysis (participation rate: 84.2%).

#### Inclusion and exclusion criteria

The inclusion criteria were as follows: being enrolled and attending classes during the study period, being a nursing student participating in clinical practice, accurately completing the questionnaire, and voluntarily agreeing to participate in the study. First-year students were excluded because they had not yet started clinical practice; therefore, the study was conducted with second-, third-, and fourth-year students.

### Data collection tools

The study data were collected using the Student Information Form, the Competency Inventory for Nursing Students, and the Management of Workplace Violence Competence Scale for Nursing Practicum Students.

#### Student information form

Developed by the researcher, the form asks about students’ sociodemographic characteristics (e.g., age, gender, academic year, self-confidence, problem-solving ability, willingness to choose the nursing department, and love for the profession) and their exposure to violence (e.g., type, time, and source of the violence). Self-confidence and problem-solving ability were measured using single-item three-category questions, while love for the profession and willingness to choose nursing were assessed using dichotomous (yes/no) items. Variables obtained from the Student Information Form were used in descriptive analyses and group comparisons, and selected variables were included as independent variables in the regression analyses.

#### Competency inventory for nursing students

Developed by Hsu and Hsieh (2013), and adapted into Turkish by Ülker and Korkmaz (2022), this 43-item, 7-point Likert-type scale includes six subscales: Clinical Biomedical Science (items 1–5), General Clinical Skills (items 6–12), Critical Thinking and Reasoning (items 13–16), Caring (items 17–22), Ethics and Responsibility (items 23–37), and Lifelong Learning (items 38–43) [20, 21]. Total scores range from 43 to 301, with higher scores indicating greater competence. The original scale’s Cronbach’s alpha ranged from 0.79 to 0.97; in this study, it was 0.96. The Turkish version demonstrated confirmed construct validity based on factor analysis [21].

#### The management of workplace violence competence scale for nursing practicum students

Originally developed by Lu et al. (2021) and adapted into Turkish by Karabey et al. (2022), this 28-item, 5-point Likert-type scale assesses nursing students’ competence in managing workplace violence [22, 23]. The scale includes four subscales consistent with the Turkish adaptation: Post-Event Recovery (items 1–10), Violence Information Management (items 11–19), Violence Response and Interaction (items 20–25), and Response to Violence (items 26–28). Scores range from 28 to 140, with higher scores indicating greater competence. The original Cronbach’s alpha was 0.88 and was 0.86 in this study. The Turkish adaptation demonstrated confirmed construct validity and strong content validity based on factor analysis [23].

All measurements were based on students’ self-reported responses using standardised instruments administered in the same format to all participants. Previously validated Turkish versions of both scales were used in this study. Internal consistency reliability was reassessed using Cronbach’s alpha coefficients for the current sample. Standardised administration procedures were applied to all participants. The Student Information Form consisted of descriptive items and did not require psychometric validation.

### Data collection

Students were accessed through the nursing department with institutional permission, and data were collected by the researchers using structured questionnaires in classroom settings. The questionnaires were completed during scheduled class hours under the supervision of the researchers. The average time required to complete the questionnaires was approximately 15–20 min. Prior to data collection, nursing students were provided with detailed information about the study. The researchers explained the types of violence and clarified the concept of violence, addressing any questions raised by the students. Informed consent was obtained from those who voluntarily agreed to participate. Questionnaires that were incomplete or contained errors were excluded from the final analysis.

### Variables

The primary outcomes of this study were nursing students’ professional competence and their violence management competence. Exposure to workplace violence and sociodemographic characteristics (age, gender, academic year, self-confidence, problem-solving ability, willingness to choose nursing and love for the profession) were examined as independent variables. Several of these factors, particularly self-confidence and problem-solving ability, were examined as descriptive characteristics potentially associated with competence levels.

### Data analysis

Data were analysed using SPSS 25.0 (IBM Corp., Armonk, NY, USA). The normality of the study variables was assessed by examining skewness and kurtosis values. Distributions with skewness and kurtosis between − 3 and + 3 are generally considered to approximate normality, thereby supporting the use of parametric statistical analyses [24]. Depending on distribution, independent group differences were examined using independent t-test or one-way ANOVA, as appropriate. Correlation and regression analyses were conducted to assess relationships while adjusting for potential confounders. Age was included as a continuous variable in regression analyses. There were no missing data, as incomplete questionnaires were excluded. The sampling strategy did not require weighting, and no sensitivity analyses were performed. To assess the assumption of multicollinearity in multiple linear regression analysis, the VIF, inter-variable correlations, and Durbin-Watson statistic were examined. It was determined that the VIF values ranged from 1.077 to 1.188 (VIF < 10; preferably < 5), the correlation coefficients between variables remained below 0.80 (*r* < 0.80), and the highest correlation was 0.384. Furthermore, the Durbin-Watson value of 1.886 (the acceptable range is 1.5–2.5) indicates that there is no autocorrelation among the error terms. These findings reveal that there is no situation in the model that indicates a problem of multicollinearity.

To minimise reporting and social desirability bias, anonymous self-administered questionnaires were used. Clear definitions and examples of workplace violence were provided before data collection to ensure accurate understanding of the items.

## Results

Detailed descriptive statistics and subscale-level results are presented in the tables; therefore, the Results section focuses on summarising key patterns and statistically meaningful findings rather than reproducing all tabulated details.

A total of 223 nursing students participated in the study (mean age 21.16 ± 1.61 years; 65% female). More than half reported high self-confidence and successful problem-solving ability; however, 45.8% had experienced workplace violence during clinical practice. Verbal and psychological violence were the most frequently reported forms, most commonly perpetrated by patients or their relatives, followed by nurses (Table 1).

**Table 1:** Sociodemographic characteristics and exposure to violence among nursing students (*n* = 223)

Characteristics		Mean	SD
Age		21.16	1.6
		**n**	**%**
Gender	Female Male	145 78	65.0 35.0
Grade level	2 3 4	74 73 76	33.2 32.7 34.1
Self-confidence	Always Sometimes Never	124 93 6	55.6 41.7 2.7
Problem-solving ability	Successful Partly Unsuccessful	118 102 3	52.9 45.7 1.3
Choosing nursing willingly	Yes No	88 135	39.5 60.5
Love for the nursing profession	Yes No	125 98	56.1 43.9
Exposure to violence during clinical practice	Yes No	102 121	45.8 54.2
Violence-related characteristics among students exposed to violence (*n* = 102)			
In which grade did you experience violence? ^*^	1 2 3 4	22 60 48 12	21.6 58.8 47.1 11.8
Type of violence experienced^*^	Physical violence Emotional violence Verbal violence Psychological violence Gender discrimination	3 34 64 62 18	2.9 33.3 62.7 60.8 17.6
Perpetrators of violence^*^	Patient/relatives Nurse Instructor Physician Classmate Other	73 48 19 6 3 4	71.6 47.1 18.6 5.9 2.9 3.9
Violence intervention situation	Yes No	46 56	45.1 54.9
What did you do when you were subjected to violence? ^*^	I told it to a friend or people I trusted I told the incident to my responsible lecturer I told my parents I tried to solve the problem with the relevant person I didn’t do anything.	33 26 1 24 18	32.4 25.5 1.0 23.5 17.6

^*^Multiple responses were allowed for violence-related items; therefore, the total number of responses exceeds the number of exposed students (*n* = 102)

Students’ mean total score on the inventory measuring professional competence was 231.65 ± 37.14, indicating a competence level slightly above the moderate range. Based on mean item scores, the highest competency level was observed in the ethics and responsibility subscale, whereas the lowest was in the clinical biomedical science subscale. Similarly, the mean total score on the Management of Workplace Violence Competence Scale for Nursing Practicum Students was 98.23 ± 13.48, reflecting a level of violence-management competence slightly above the moderate range. According to mean item scores, the highest subscale score was obtained in violence response and interaction, while the lowest was observed in response to violence (Table 2).

**Table 2:** Mean scores of nursing students on the competency scale and the management of workplace violence competence scale (*n* = 223)

**Competency Inventory for Nursing Students**	**Skewness**	**Kurtosis**	**Mean ± SD**	**Mean per Item** **(1–7)**
Clinical biomedical science subscale General clinical skills subscale Critical thinking and reasoning subscale Care subscale Ethics and responsibility subscale Lifelong learning subscale Total score (min: 43, max: 301)	-0.323 -0.474 -0.134 -0.487 -1.427 -0.997 -0.773	0.429 -0.192 -0.015 -0.189 2.538 0.979 0.822	21.86 **±** 5.90 35.85 ± 8.29 19.58 ± 4.45 32.47 ± 6.61 88.95 ± 14.05 33.06 ± 6.93 231.65 **±** 37.14	4.37 5.12 4.89 5.41 5.93 5.51 5.38
**Management of Workplace Violence Competence Scale for Nursing Practicum Students**	**Skewness**	**Kurtosis**	**Mean ± SD**	**Mean per Item** **(1–5)**
Post-event recovery subscale Violence information management subscale Violence response and interaction subscale Response to violence subscale Total score (min: 28, max: 140)	-1.176 -0.423 -0.683 -0.118 -0.692	2.667 1.453 2.528 -0.097 2.250	36.87 ± 6.04 29.71 ± 4.99 22.22 ± 3.39 9.42 ± 2.44 98.23 ± 13.48	3.68 3.30 3.70 3.14 3.50

SD: Standard Deviation

Professional competence scores varied significantly by several sociodemographic factors including gender, academic year, self-confidence, problem-solving ability and motivation-related variables, with higher scores generally observed among females, upper-year students and those with stronger self-confidence and problem-solving skills (Table 3).

**Table 3:** Comparison of competence inventory scores across sociodemographic characteristics of nursing students (*n* = 223)

Characteristics		Total Mean ± SD	Clinical biomedical science subscale Mean ± SD	General clinical skills subscale Mean ± SD	Critical thinking and reasoning subscale Mean ± SD	Care subscale Mean ± SD	Ethics and responsibility subscale Mean ± SD	Lifelong learning subscale Mean ± SD
Gender	Female Male	236.61 ± 34.31 222.51 ± 40.53	22.16 ± 6.06 21.16 ± 5.57	37.25 ± 7.77 33.24 ± 8.63	19.84 ± 4.39 19.10 ± 4.56	33.56 ± 6.03 30.46 ± 7.19	90.46 ± 11.06 86.16 ± 17.06	33.50 ± 6.39 32.24 ± 7.80
	t p	**2.73** **0.007**	1.078 0.29	**3.533** **0.001**	1.192 0.23	**3.420** **0.001**	**2.195** **0.02**	1.297 0.24
	Cohen’s d	**0.39**	-	**0.50**	-	**0.48**	**0.31**	-
Grade level	2 3 4	220.64 ± 39.78 237.97 ± 34.35 236.37 ± 35.05	19.89 ± 5.63 23.43 ± 5.36 22.26 ± 6.17	33.17 ± 8.72 37.19 ± 8.32 37.17 ± 7.22	18.72 ± 4.67 20.20 ± 4.06 19.93 ± 4.50	31.36 ± 7.13 33.75 ± 5.87 32.33 ± 6.65	85.97 ± 16.07 90.10 ± 12.35 90.37 ± 13.16	31.62 ± 7.74 33.27 ± 6.22 34.26 ± 6.55
	F p	**5.09** **0.007**	**7.294** **0.001**	**6.063** **0.003**	2.708 0.069	2.452 0.089	2.577 0.078	2.819 0.062
	η²	**0.04**	**0.06**	**0.05**	-	-	-	-
Self-confidence	Always Sometimes Never	237.79 ± 37.96 227.00 ± 31.79 178.00 ± 49.48	22.65 ± 6.06 21.16 ± 5.36 16.33 ± 7.08	36.66 ± 8.44 35.33 ± 7.60 27.00 ± 7.88	20.47 ± 4.35 18.78 ± 4.01 13.66 ± 6.97	33.38 ± 6.40 31.84 ± 6.51 25.66 ± 8.71	90.17 ± 14.47 88.13 ± 12.46 76.50 ± 20.92	34.75 ± 6.46 31.73 ± 5.99 18.83 ± 9.84
	F p	**16.21** **0.001**	**4.63** **0.01**	**6.64** **0.03**	**15.29** **0.001**	**7.64** **0.02**	**6.01** **0.04**	**28.63** **0.01**
	η²	**0.07**	**0.04**	**0.04**	**0.08**	**0.04**	**0.03**	**0.02**
Problem-solving ability	Successful Partly Unsuccessful	235.03 ± 37.03 229.73 ± 34.68 163.70 ± 34.68	22.44 ± 5.95 21.35 ± 5.66 16.00 ± 9.53	36.34 ± 8.21 35.60 ± 8.07 24.66 ± 13.57	20.06 ± 4.30 19.30 ± 4.32 10.33 ± 6.02	32.97 ± 6.66 32.20 ± 6.30 22.00 ± 8.18	89.33 ± 14.51 89.02 ± 13.10 71.66 ± 21.57	33.85 ± 6.32 32.55 ± 6.87 19.00 ± 16.09
	F p	**6.65** **0.03**	4.28 0.11	3.42 0.18	**8.96** **0.01**	**5.69** **0.05**	3.42 0.16	5.12 0.07
	η²​	**0.05**	-	-	**0.07**	**0.04**	-	-
Choosing nursing willingly	Yes No	239.80 ± 31.70 226.30 ± 39.53	22.10 ± 5.79 21.70 ± 5.98	37.46 ± 7.28 34.80 ± 8.75	20.47 ± 4.19 19.00 ± 4.54	34.01 ± 5.42 31.47 ± 7.13	90.67 ± 12.23 87.84 ± 15.06	35.07 ± 5.56 31.74 ± 7.41
	t p	**2.686** **0.008**	0.49 0.62	**2.371** **0.01**	**2.475** **0.01**	**2.845** **0.005**	1.537 0.12	**3.875** **0.001**
	Cohen’s d	**0.37**	-	**0.33**	**0.34**	**0.39**	-	**0.53**
Love for the nursing profession	Yes No	240.60 ± 30.76 220.33 ± 41.38	22.88 ± 4.80 20.55 ± 6.86	37.35 ± 7.41 33.93 ± 8.97	20.10 ± 3.95 18.90 ± 4.97	34.03 ± 5.52 30.51 ± 7.36	91.66 ± 11.48 85.51 ± 16.18	34.74 ± 5.63 30.91 ± 7.81
	t p	**4.185** **0.001**	**2.987** **0.003**	**3.110** **0.002**	**2.028** **0.04**	**4.074** **0.001**	**3.317** **0.001**	**4.085** **0.001**
	Cohen’s d	**0.56**	**0.40**	**0.42**	**0.27**	**0.55**	**0.45**	**0.55**

SD: Standard Deviation, t: Student t-test, F: ANOVA. Effect sizes are reported as Cohen’s d for t-tests and eta-squared (η²) for ANOVA. Cohen’s d values of 0.20, 0.50, and 0.80 indicate small, medium, and large effects, respectively; η² values of 0.01, 0.06, and 0.14 indicate small, medium, and large effects

Violence management competence scores varied significantly by several factors, including self-confidence and problem-solving ability, with higher scores generally observed among those reporting stronger self-confidence and better problem-solving skills (Table 4).

**Table 4:** Comparison of nursing students’ workplace violence management competence scores based on sociodemographic characteristics (*n* = 223)

Characteristics		Total Mean ± SD	Post-event recovery subscale Mean ± SD	Violence information management subscale Mean ± SD	Violence response and interaction subscale Mean ± SD	Response to violence subscale Mean ± SD
Gender	Female Male	98.09 ± 13.04 97.00 ± 14.25	37.31 ± 5.86 36.03 ± 6.32	29.93 ± 4.79 29.31 ± 5.35	22.29 ± 3.44 22.08 ± 3.31	9.34 ± 2.42 9.56 ± 2.48
	t p	1.005 0.31	1.510 0.13	0.899 0.37	0.433 0.66	0.613 0.54
	Cohen’s d	-	-	-	-	-
Grade level	2 3 4	97.75 ± 11.98 97.91 ± 14.85 99.01 ± 13.63	37.36 ± 5.52 36.26 ± 6.54 36.80 ± 6.06	29.89 ± 4.74 30.10 ± 5.07 29.69 ± 5.17	21.59 ± 2.97 22.18 ± 4.08 22.88 ± 2.94	9.44 ± 2.25 9.36 ± 2.57 9.46 ± 2.51
	F p	0.192 0.82	0.628 0.53	0.423 0.65	2.747 0.06	0.029 0.97
	η²	-	-	-	-	-
Self-confidence	Always Sometimes Never	100.10 ± 14.57 96.94 ± 11.06 79.66 ± 8.01	37.82 ± 6.11 36.18 ± 5.54 27.83 ± 2.92	30.19 ± 5.19 29.41 ± 4.54 24.50 ± 4.76	22.61 ± 3.75 21.83 ± 2.85 20.15 ± 1.94	9.47 ± 2.70 9.50 ± 2.02 7.16 ± 1.94
	** F** p	**15.28** **0.001**	**17.62** **0.01**	**7.39** **0.02**	**10.32** **0.01**	**5.29** **0.05**
	η²	**0.07**	**0.08**	**0.04**	**0.03**	**0.03**
Problem-solving ability	Successful Partly Unsuccessful	101.03 ± 12.04 95.39 ± 14.14 85.00 ± 21.63	37.96 ± 5.08 35.81 ± 6.73 29.66 ± 7.23	30.71 ± 4.67 28.70 ± 4.98 25.00 ± 9.53	22.62 ± 3.38 21.82 ± 3.39 20.66 ± 2.08	9.74 ± 2.47 9.04 ± 2.36 9.66 ± 3.05
	** F** p	**7.41** **0.02**	**7.73** **0.02**	**8.28** **0.01**	4.11 0.12	4.13 0.12
	η²	**0.06**	**0.05**	**0.05**	-	-
Choosing nursing willingly	Yes No	100.20 ± 13.14 96.95 ± 13.60	37.78 ± 5.39 36.27 ± 6.38	30.39 ± 5.02 29.27 ± 4.94	22.51 ± 3.20 22.03 ± 3.51	9.51 ± 2.44 9.37 ± 2.44
	t p	1.766 0.079	1.832 0.06	1.644 0.10	1.020 0.30	0.475 0.67
	Cohen’s d	-	-	-	-	-
Love for the nursing profession	Yes No	99.72 ± 13.28 96.34 ± 13.57	37.76 ± 5.78 35.73 ± 6.21	29.96 ± 4.84 29.39 ± 5.18	22.38 ± 3.29 22.02 ± 2.50	9.60 ± 2.50 9.19 ± 2.35
	t p	1.865 0.06	**2.511** **0.01**	0.847 0.39	0.793 0.42	1.258 0.20
	Cohen’s d	-	**0.34**	-	-	-

SD: Standard Deviation, t: Student’s t-test, F: ANOVA. Effect sizes are reported as Cohen’s d for t-tests and eta-squared (η²) for ANOVA. Cohen’s d values of 0.20, 0.50, and 0.80 indicate small, medium, and large effects, respectively; η² values of 0.01, 0.06, and 0.14 indicate small, medium, and large effects

A moderate positive correlation was identified between nursing students’ professional competence levels and their violence management competence in clinical practice (*r* = 0.384, *p* = 0.001, Table 5).

**Table 5:** The relationship between the workplace violence management competence scale and the competence inventory for nursing students (*n* = 223)

		1	2	3	4	5	6	7	8	9
**1** MWVCS Total	r	1								
	p	**-**								
**2** CINS Total	r	0.384	1							
	p	**0.001**	-							
**3** Clinical biomedical science subscale	r	0.273	0.621	1						
	p	**0.001**	**0.010**	-						
**4** General clinical skills subscale	r	0.345	0.857	0.601	1					
	p	**0.001**	**0.010**	**0.010**	-					
**5 **Critical thinking and reasoning subscale	r	0.357	0.789	0.498	0.726	1				
	p	**0.001**	**0.010**	**0.010**	**0.010**	-				
**6** Care subscale	r	0.303	0.883	0.320	0.585	0.619	1			
	p	**0.010**	**0.010**	**0.010**	**0.010**	**0.010**	-			
**7** Ethics and responsibility subscale	r	0.243	0.772	0.375	0.637	0.567	0.608	1		
	p	**0.001**	**0.010**	**0.010**	**0.010**	**0.010**	**0.010**	-		
**8** Lifelong learning subscale	r	0.387	0.871	0.353	0.569	0.555	0.582	0.646	1	
	p	**0.010**	**0.010**	**0.010**	**0.010**	**0.010**	**0.010**	**0.010**	-	
**9** Age	r	0.025	0.100	0.104	0.080	0.119	0.072	0.058	0.098	1
	p	0.705	0.136	0.122	0.236	0.076	0.288	0.392	0.144	-

r = Pearson Correlation, MWVCS: Management of workplace violence competence scale for nursing practicum students, CINS: Competency inventory for nursing students

The multiple regression model examining the association between nursing students’ professional competence and violence management competence was statistically significant (F(6, 214) = 5.563, *p* < 0.001) and explained 15.4% of the variance in violence management competence (R² = 0.154). Professional competence (CINS total score) was a significant positive predictor of violence management competence (unstandardised β = 0.141, SE = 0.025, standardised β = 0.388, t = 5.665, *p* < 0.001). Age, gender, grade level, choosing nursing willingly, and love for the nursing profession were not significantly associated with violence management competence (*p* > 0.05). These findings suggest that professional competence independently predicts students’ ability to manage workplace violence in clinical practice, after adjusting for age, gender, grade level, choosing nursing willingly, and love for the nursing profession (Table 6).

**Table 6:** Regression analysis of nursing students’ professional competence and violence management competence in clinical practice (*n* = 223)

	Unstandardised β	SE	Standardised Coefficients β	t	*p*	95% CI
Constant	65.602	13.470		4.870	**< 0.001**	[39.050–92.153]
Independent variable (CINS total)	0.141	0.025	0.388	5.665	**< 0.001**	[0.092–0.190]
Age	0.079	0.620	0.009	0.128	0.898	[-1.142–1.300]
Gender	0.025	1.842	0.001	0.013	0.989	[-3.607–3.656]
Grade level	-0.998	2.402	-0.035	-0.415	0.678	[-5.731–3.736]
Choosing nursing willingly	-1.099	1.976	-0.040	-0.556	0.579	[-4.993–2.795]
Love for the nursing profession	-0.041	1.989	-0.002	-0.020	0.984	[-3.961–3.879]

Dependent variable: Management of workplace violence competence scale for nursing practicum students. Independent variable: Competency inventory for nursing students. Model fit: *R* = 0.392, R² = 0.154, F(6,214) = 5.563, *p* < 0.001. SE: Standard Error. Categorical variables were dummy coded; reference categories were female, 2nd year, and ‘yes’

## Discussion

This study indicates a moderate positive relationship between professional competence and violence management competence, suggesting that students who feel more competent in their clinical role may be better prepared to recognise, respond to and recover from violent situations. Professional competence is commonly associated with core capabilities, such as communication, situational awareness, and ethical judgement, that can help prevent escalation and support safer interactions [11, 12, 25]. Although clinical placements are shown to enhance professional competence through supervised learning and exposure to real care environments [26], there remains limited evidence on how these competencies specifically translate into violence-management capability. To our knowledge, this study is among the limited number of studies examining the direct association between professional competence and violence management competence among nursing students, and it extends existing evidence by highlighting this relationship within undergraduate clinical education. From a mechanistic perspective, this relationship may be explained by self-efficacy. Students with higher professional competence are likely to feel more confident in managing difficult interpersonal situations and to use more effective problem-solving strategies during violent encounters. Although the strength of the association was moderate, this finding suggests that even modest differences in professional competence may be associated with meaningful differences in nursing students’ ability to cope with violent situations in clinical practice. Given the cross-sectional design and shared variance among key constructs, including professional competence, self-confidence, and problem-solving ability, the observed associations should be interpreted as relational rather than causal. In line with the cross-sectional design and study objectives, the analyses should be considered exploratory in nature and intended to identify patterns of association rather than to test causal hypotheses.

This study showed that nursing students with higher levels of self-confidence and stronger problem-solving ability demonstrated significantly greater professional competence. Similar findings have been reported in previous studies, indicating that these characteristics support students’ engagement in learning and clinical performance [7, 10, 16, 27]. Students who willingly chose the nursing profession also exhibited higher competence levels, suggesting that intrinsic motivation contributes positively to professional development. However, some studies have reported conflicting results, indicating that self-confidence and motivation may not always translate directly into higher professional competence, particularly in the presence of inadequate clinical support or high academic stress [16, 17, 28]. These findings highlight the importance of educational strategies that strengthen self-confidence, problem-solving skills, and professional motivation during nursing education.

In this study, nearly half of the nursing students reported experiencing workplace violence during clinical placements, with verbal and psychological abuse most commonly perpetrated by patients, relatives and occasionally nurses. This finding is consistent with previous studies reporting that nursing students frequently encounter non-physical forms of aggression in clinical environments [4, 6–8, 10]. The high prevalence observed in the present study highlights the continued impact of workplace violence on students’ wellbeing and learning experiences and underscores the need for stronger preventive strategies within nursing education and institutional policy. This finding is supported by recent qualitative evidence showing the negative emotional and professional impact of workplace violence on nursing students [14]. Cultural and social factors may play an important role in shaping these findings within the Turkish clinical context. High patient density, heavy workloads, and hierarchical relationships in healthcare settings may increase the risk of aggressive interactions [6, 7, 10]. In addition, cultural norms related to authority and communication may discourage students from reporting violent incidents or seeking institutional support, which may partially explain the high prevalence observed in this study [3, 4].

The findings of this study should also be interpreted within the specific context of the Turkish healthcare and nursing education systems. High patient density, heavy clinical workloads, and hierarchical organisational structures in healthcare settings may increase nursing students’ exposure to aggressive behaviours and shape their opportunities to develop professional competence during clinical training. Similar contextual challenges have been reported in other countries; however, differences in educational models, clinical supervision, and institutional support mechanisms may influence both the prevalence of workplace violence and students’ coping capacities. These contextual factors should be considered when comparing the present findings with international studies [3, 4, 6, 7, 9, 15, 29].

Previous research identifies patients, their relatives and, at times, nurses as the main perpetrators of workplace violence against nursing students [4, 6–8]. Such incidents are known to adversely affect students’ clinical learning, self-confidence and willingness to engage in patient care [6]. The concentration of violent experiences in the later years of training could be attributed to increased exposure to direct patient care, communication demands and students’ growing responsibility in clinical decision-making. While senior students may possess more advanced coping strategies, they are also placed in more complex and demanding environments, potentially heightening their vulnerability to aggression [6, 7]. These findings demonstrate the necessity for structured interventions, including violence-prevention education, enhanced supervisory support and institutional reporting systems that protect students and foster a safe learning environment.

In this study, nursing students demonstrated professional competence levels that were slightly above the moderate range across all subscales of the Competency Inventory for Nursing Students. Similar outcomes have been reported in studies that used the same instrument in different educational contexts [17, 18, 21, 28], suggesting that core competency development is progressing positively among nursing students in Türkiye. Structured curricula, supervised clinical learning, and competency-based teaching strategies may all contribute to these results. However, as competence levels remain moderately high, further enhancement is needed in promoting students’ readiness for complex clinical situations and increasing opportunities for skills consolidation throughout clinical training.

In this study, nursing students displayed competence levels slightly above the moderate range in managing workplace violence, which aligns with previous findings from similar studies [3, 6–8]. However, conflicting evidence has also been reported, with some research suggesting that students feel inadequately prepared to cope with violent incidents [4]. These inconsistencies likely reflect differences in violence-prevention education, the emphasis placed on communication and de-escalation strategies within curricula, and students’ confidence in reporting violent experiences [7, 9, 15]. Despite moderately positive outcomes, these results remain insufficient given the frequency and severity of violence in clinical settings. Therefore, comprehensive and proactive training approaches are needed to strengthen students’ ability to recognise, respond to and report aggression effectively.

In this study, professional competence was significantly higher among female students, upper-year students, and those with greater self-confidence, stronger problem-solving ability, and higher professional motivation. Similar patterns have been reported in previous studies, indicating that both educational progression and individual psychological resources contribute to competence development [16, 17, 28]. These findings highlight the importance of structured, progressive clinical education and the need to support students’ self-confidence, problem-solving skills, and professional motivation throughout undergraduate training.

In this study, gender and enthusiasm for the nursing profession showed minimal influence on students’ competence in managing workplace violence, which is consistent with previous studies demonstrating limited demographic effects [3, 6]. These findings suggest that violence management competence is shaped less by personal characteristics and more by clinical training, exposure to real incidents and institutional support. Although a significant association was observed only between love for the profession and post-event recovery, the literature indicates that exposure to violence can erode students’ confidence in the profession and diminish their commitment to a nursing career [15, 29]. Failure to address the emotional burden of violence may weaken motivation and hinder the acquisition of coping strategies over time. This underscores the need for systematic violence-prevention education and supportive clinical supervision to protect students’ psychological wellbeing and sustain their professional identity.

In this study, higher levels of self-confidence and stronger problem-solving ability were descriptively associated with greater competence in managing workplace violence, suggesting that psychological readiness may be relevant to students’ responses to aggression. This finding aligns with previous research demonstrating that confidence and problem-solving support the development of professional identity and effective clinical decision-making [30, 31]. Students who perceive themselves as competent may be more willing to intervene, utilise de-escalation strategies and seek assistance when needed. Therefore, educational approaches that support students’ psychological readiness, such as simulation-based education, reflective practice and communication training, may enhance students’ preparedness to cope with workplace violence.

Overall, the findings indicate that nursing education should simultaneously prioritise competence development and violence-prevention strategies, as both are interdependent components of safe and effective clinical practice. These results should be interpreted with caution due to the cross-sectional design and the multiple comparisons performed. Furthermore, although the findings are consistent with previous research, they require confirmation in larger and more diverse populations. Considering these findings, nursing curricula could integrate structured simulation scenarios, de-escalation and communication skills training, and reflective practice sessions to strengthen students’ preparedness for violent situations. Embedding such strategies within competence-based education may support the development of both professional competence and violence-management abilities. This study adds to the existing literature by demonstrating an association between professional competence and violence management competence among nursing students, suggesting that professional competence may play a potential protective role within undergraduate nursing education.

### Limitations of the study

This study has several limitations related to measurement, sampling, and statistical modelling. It was conducted in a single institution and employed a cross-sectional design, which limits the generalisability of the findings and precludes causal inference. Consequently, temporal relationships between variables cannot be established. Data were collected using self-report measures, which may be subject to response and recall bias, particularly regarding experiences of workplace violence. Reliance on self-reported measures may have introduced social desirability bias or overestimation of students’ perceived competencies, particularly regarding professional competence and violence management. Although anonymous data collection was used to mitigate this risk, self-reported perceptions may not fully reflect actual clinical behaviour. Self-confidence and problem-solving ability were assessed using single-item measures, which may limit conceptual and analytical precision. Future research should employ validated multi-item scales and incorporate observational assessments, supervisor evaluations, or mixed-methods designs to complement self-reported data. Additionally, important institutional and contextual factors such as the type of clinical unit in which violence occurred, the level of support provided by clinical instructors, and students’ prior exposure to institutional violence prevention policies and training were not assessed. These unmeasured factors may have influenced students’ perceptions and perceived competence in managing violent incidents. Therefore, the findings of this study should be interpreted with caution regarding their generalisability to other institutional and cultural contexts. In addition, workplace violence exposure was assessed only as a binary variable (yes/no); therefore, the frequency and severity of violent experiences could not be examined in this study. The lack of information on the frequency and severity of violence may have limited more nuanced interpretation of the findings.

Accordingly, the findings should be interpreted strictly as relational associations rather than evidence of independent, causal, or directional relationships between professional competence and violence management competence. In addition, both professional competence and violence management competence were assessed using self-report scales that share partially overlapping theoretical domains, such as communication, responsibility, and critical thinking. This conceptual overlap may have contributed to the observed association and should be considered when interpreting the strength and novelty of the findings.

Beyond confirming an association between professional competence and violence management competence, this study conceptually positions professional competence as a potential protective and enabling factor in undergraduate nursing education. By framing professional competence within a self-efficacy perspective, the findings suggest that stronger core professional competencies may be associated with greater preparedness to recognise, manage, and recover from workplace violence during clinical practice.

## Conclusions

Approximately half of the students reported exposure to violence during clinical practice, most commonly verbal or psychological in nature and often perpetrated by patients, their relatives or nurses. Students demonstrated competence levels slightly above moderate in both professional competence and violence management, and violence management competence was positively associated with professional competence. Gender, year of study, motivation for choosing nursing and commitment to the profession were associated with professional competence, while self-confidence and problem-solving ability showed descriptive associations based on single-item self-reports. Overall, a moderate positive correlation was found between professional competence and violence management competence.

These findings highlight the need for educational strategies that strengthen both competence domains. Integrating structured training such as simulation-based scenarios, de-escalation and communication skills training, and opportunities for guided reflection may enhance students’ preparedness for violent situations during clinical education and support their resilience in future professional practice. In addition, future studies using longitudinal and multi-centre designs are needed to better clarify causal relationships and improve the generalisability of these findings. Given the single-centre, cross-sectional design, these findings should be interpreted as context-specific and exploratory.

## Implications for education, practice, and policy

### Implications for education

Nursing curricula could integrate structured modules on workplace violence prevention and management, including simulation-based training on de-escalation, communication, and conflict-resolution skills. In addition, educational interventions that strengthen students’ self-efficacy—such as reflective practice, mentorship, and supervised clinical feedback—may enhance both professional competence and preparedness for managing violent situations.

### Implications for practice

Hospital administrators should establish clear protocols for reporting and managing workplace violence involving students and ensure access to psychological support and trained clinical mentors.

### Implications for policy

Despite the single-centre design, the findings point to the need for institutional and educational policies supporting the prevention and management of workplace violence against nursing students.

## Data Availability

The datasets used and analysed during the current study are available from the corresponding author on reasonable request.

## Declarations

### Use of artificial intelligence

An AI-based language model (ChatGPT, OpenAI) was used to support language editing of this manuscript. All content was reviewed and verified by the authors, who take full responsibility for the final version of the text.
